# Multivariate phenotyping reveals thermoregulatory patterns in crossbred cattle

**DOI:** 10.1007/s11250-026-05416-9

**Published:** 2026-10-18

**Authors:** Robson Mateus Freitas Silveira, Isadora Emanoela Pereira Costa Andrade, Luciano Barreto-Mendes, Larissa de Melo Coelho, Concepta McManus, Luiz Paulo Fávero, Débora Andrea Evangelista Façanha

**Affiliations:** 1 Department of Biosystems Engineering, “Luiz de Queiroz” College of Agriculture, University of São Paulo (ESALQ/USP), Av. Pádua Dias, 11 – Agronomia, Piracicaba, SP 13418-900 Brazil; 2 MBA in Data Science and Analytics, Luiz de Queiroz College of Agriculture, University of São Paulo (ESALQ/USP), Av. Pádua Dias, 11 – Agronomia, Piracicaba, SP 13418-900 Brazil; 3 Université Clermont Auvergne, INRAE, VetAgro Sup, UMR Herbivores, Saint-Genes Champanelle, 63122 France; 4 Department of Animal Science, “Luiz de Queiroz” College of Agriculture, University of São Paulo (ESALQ/USP), Av. Pádua Dias, 11 – Agronomia, Piracicaba, SP 13418-900 Brazil; 5 Center for Nuclear Energy in Agriculture (CENA), University of São Paulo (USP), Av. Centenário, 303 – São Dimas, Piracicaba, SP 13416-000 Brazil; 6 School of Economics, Business and Accounting (FEA), University of São Paulo (USP), Av. Prof. Luciano Gualberto, 908 – Butantã, São Paulo, SP 05508-010 Brazil; 7 Federal University of Ceará (UFC), Av. Mister Hull, 2977 – Pici Campus, Fortaleza, CE 60440-554 Brazil

**Keywords:** Exploratory phenotyping, Coat traits, Heat dissipation, Tropical climate, Resilience

## Abstract

Improving the adaptation of beef cattle production to climate change requires crossbreeding strategies that enhance the ability of animals to maintain thermal stability under hot conditions. The objective of this exploratory study was to assess how coat characteristics are associated with rectal-temperature variation and to identify thermoregulatory patterns in Red Angus × Nelore crossbred cattle raised under tropical conditions. Thirty clinically healthy crossbred bulls from two genetic compositions (½ Red Angus × ½ Nelore and ¾ Red Angus × ¼ Nelore) were evaluated at 210 and 360 days of age. Morphological, physiological, and environmental variables were recorded, including coat thickness, hair length, hair quantity, hair density, and rectal temperatures measured in the morning and afternoon. Univariate comparisons did not detect differences (*p* > 0.05) between the genetic groups in the evaluated coat traits or rectal temperatures, indicating substantial overlap between genetic compositions. In an exploratory logistic model developed to discriminate the two genetic groups, rectal temperature was positively associated with group classification [odds ratio (OR) = 1.41, *p* = 0.023], whereas hair length showed a negative association (OR = 0.53, *p* < 0.001), indicating that morphological and physiological traits jointly contributed to the observed differentiation. Correlation analysis showed that thicker coats tended to contain fewer and less densely distributed hairs, whereas hair quantity and density were strongly and positively associated, suggesting compensatory coat architectures. The exploratory clustering solutions suggested four candidate coat-related configurations: (*i*) short, dense coats and (*ii*) thick but less dense coats, both associated with lower daily rectal-temperature variation; and (*iii*) intermediate coats and (*iv*) long, dense coats, associated with greater thermal fluctuation. Although some overlap among clusters was observed, these configurations provide an exploratory basis for understanding how combinations of coat characteristics may influence thermoregulatory responses. The findings indicate that thermal regulation in crossbred cattle is better understood through combinations of morphological traits than through isolated characteristics. Future studies with larger, independent populations and additional physiological and productive measurements are needed to confirm the consistency and broader applicability of these patterns.

**Graphical Abstract:**

**Figure:**
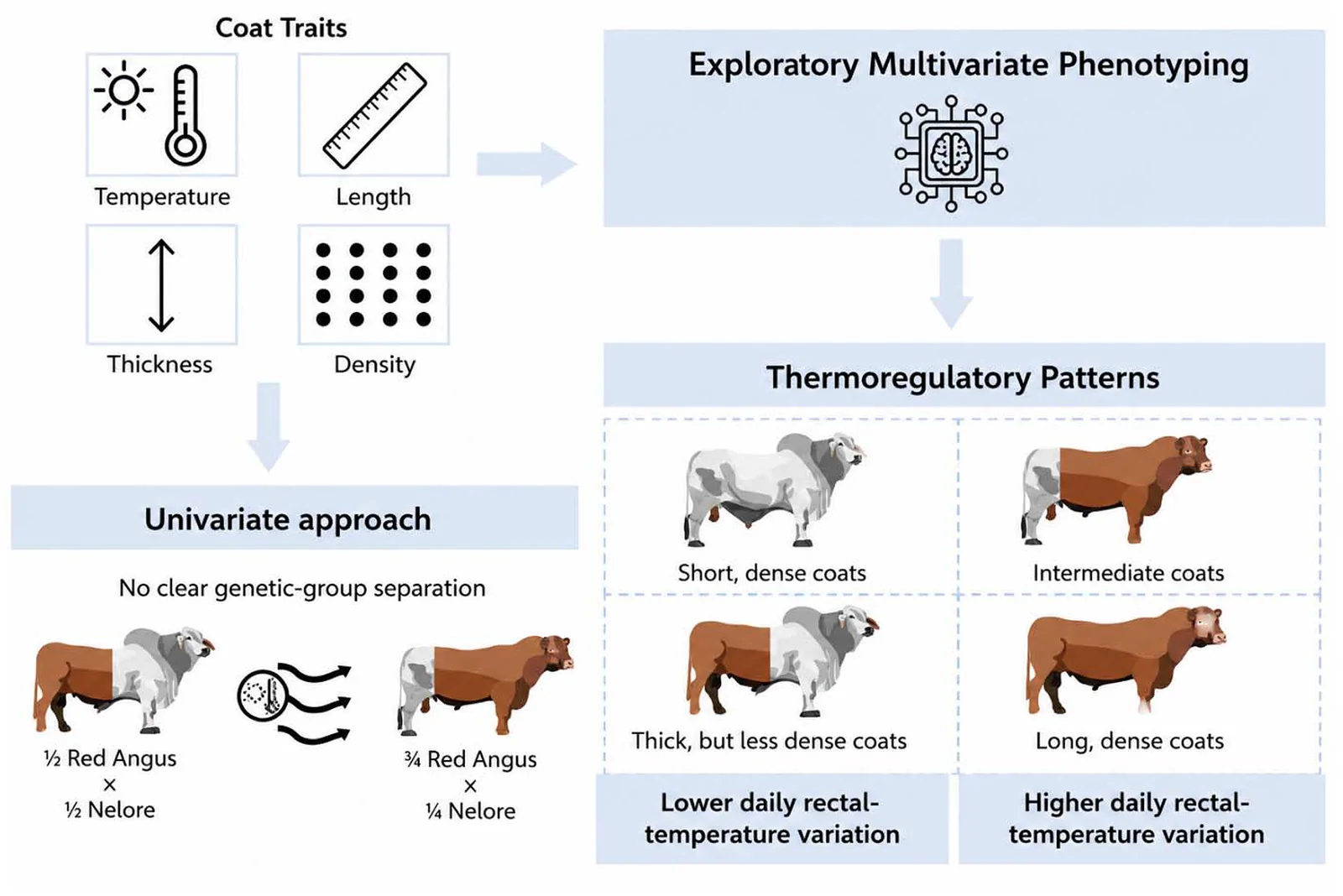

## Introduction

The growing global demand for beef imposes pressure on this type of system, particularly in tropical regions where excessive heat threatens productivity (Silveira et al. 2026). This challenge is exacerbated by the climate change crisis, which increases the frequency and intensity of heat stress events. Brazil, the world’s largest beef exporter and second-largest producer (FAO 2023), addresses this challenge by, among other strategies, combining high-yield breeds with lineages adapted to tropical climate.

Crossbreeding between taurine and zebuine cattle is widely used to capture heterosis for growth and carcass quality without compromising their capacity of quickly dissipating heat in case of a heat wave, i.e. increasing their heat resilience, hereby defined as the ability to maintain thermal homeostasis and physiological stability under acute heat stress in tropical environments (Sejian et al. 2018). Nevertheless, the performance of crossbreeds can be limited by the lower heat tolerance of European breeds under hot climates (Michael et al. 2022). The heritability of thermoregulatory traits, such as rectal temperature, is moderate; however, their phenotypic expression is strongly modulated by environment and by animal morphology (Hernandez et al. 2024).

Heat dissipation is driven primarily by evaporation (sweating and panting) and, to a lesser extent, by conduction and radiation. Zebu (*Bos indicus*) breeds typical of the tropics, such as Nelore (Bos taurus indicus), display body attributes that favor heat transfer shorter and less dense coats, higher relative surface area, and larger sweat glands (Silveira et al. 2021). In contrast, taurine breeds from temperate climates generally have thicker, more compact coats, higher metabolic rates, and reduced heat dissipation capacity, making them more susceptible to heat stress in hot environments (Brown-Brandl, 2018; Camargo et al. 2023). For instance, Nelore cattle exhibit significantly finer coats and higher sweat-gland density than 5/8 Bos taurus × 3/8 Bos indicus crosses (Barreto et al. 2024).

Extensive evidence in ruminant thermal biology indicates that coat characteristics—such as length, thickness, density, pigmentation, and skin features—play a central role in heat exchange and thermoregulatory efficiency in production animals raised in hot environments. However, despite this consolidated body of knowledge, relatively little is known about how genetic recombination in crossbred animals reshapes coat phenotypes and modifies their functional relevance for heat dissipation under tropical and semi-arid conditions. Crossbreeding programs can generate wide phenotypic variability in coat traits, potentially producing animals with contrasting thermoregulatory capacities even within the same production system (Brown-Brandl 2018; Silveira et al. 2021; Hernandez et al. 2024). Based on this framework, we hypothesized that crossbred animals exhibit distinct coat morphologies arising from genetic recombination and that this phenotypic variability differentially modulates sensible and latent heat dissipation, resulting in measurable differences in core temperature stability and overall thermoregulatory efficiency under tropical conditions.

Against this backdrop, multivariate approaches may enable the concomitant assessment of physiological, environmental, and genetic indicators in order to comprehensively assess heat tolerance. Multivariate models have been used to predict thermal states in cattle by integrating environmental variables, such as air temperature, relative humidity, the temperature–humidity index, and the black globe humidity index, with physiological responses (Silveira et al. 2023; da Silva et al. 2025). Previous studies have shown that multivariate approaches can integrate physiological, morphological, environmental, and genetic information to characterize thermal responses in livestock (Silveira et al. 2024). By examining multiple traits simultaneously, these approaches complement traditional univariate analyses and may reveal thermoregulatory patterns that would otherwise remain obscured. Integrating physiological responses, genetic composition, and coat characteristics can provide a more comprehensive understanding of heat adaptation and may inform future phenotypic screening, breeding, and management strategies in tropical production systems.

In this context, we used an exploratory multivariate framework to assess how coat characteristics are associated with heat dissipation and rectal-temperature stability in Red Angus × Nelore crossbred cattle raised under tropical conditions. By integrating low-cost morphophysiological measurements with dimensionality-reduction and clustering techniques, this approach was designed to identify coat-related thermoregulatory patterns and to investigate compensatory relationships among morphological and physiological traits. The resulting patterns provide a biologically grounded basis for understanding thermal responses in crossbred cattle and may support future research on phenotypic screening and heat-stress management after validation in larger, independent populations. The Red Angus × Nelore cross was selected because it combines a taurine breed with high productive potential but comparatively lower heat tolerance with Nelore, a zebuine breed widely recognized for tropical adaptation. The two genetic compositions evaluated herein therefore provide a biologically relevant gradient of taurine and zebuine inheritance for exploring coat-related thermoregulatory variation under tropical conditions.

## Materials and methods

This study combined experimental and computational approaches to investigate heat-adaptation traits in crossbred cattle. Data were collected from thirty Red Angus × Nelore bulls reared under tropical feedlot conditions in northeastern Brazil, including morphological (coat traits), physiological (rectal temperature), and environmental variables.

Analyses were conducted in two stages:

i. Univariate comparisons to evaluate between-group differences in coat and temperature traits;
ii. Exploratory multivariate analyses, including dimensionality reduction and unsupervised clustering, to characterize coat-related thermoregulatory patterns.

Further detail on the description of the experimental conditions, data collection and choices of analyses is provided below.

### Study site, animals, and management conditions

The trial was carried out on a commercial farm in Ceará-Mirim, Rio Grande do Norte, Brazil (05°38′02″ S; 35°25′33″ W). The regional climate is hot semi-arid, classified as BSh according to the Köppen climate classification, with a rainy season typically occurring between March and July (Alvares et al. 2013).

A total of 30 clinically healthy bulls from two genetic groups (*n* = 15 each) [(½ Red Angus × ½ Nelore (NEL) and ¾ Red Angus (RED) × ¼ Nelore] were used. Dams were of similar age, hormonally synchronized, and artificially inseminated with conventional semen; all calves were born within a two-day interval. During the cow–calf phase, calves stayed with their dams under natural suckling and rotational grazing on Brachiaria grass (*Brachiaria brizantha*) and Mombasa grass (*Panicum maximum*) until approximately seven months of age, when they were weaned and moved directly to the feedlot, remaining there until slaughter at 15 months.

The finishing diet included elephant grass, Mombasa silage, barley, cottonseed, and a protein–mineral supplement. Throughout finishing, animals received a ration formulated to 12% crude protein, offered *ad libitum* in two daily meals, with free access to water. The diet met nutritional requirements for growing/finishing beef cattle targeting 1.3–1.6 kg/day average daily gain, according to NRC (1996). In both phases (cow–calf and finishing), the genetic groups were kept under identical housing and feeding management conditions.

### Environmental, physiological, and hair-coat measurements

The present study considered two evaluations conducted when the animals were 210 and 360 days old. The first evaluation was performed in January, at approximately 210 days of age, corresponding to the weaning period, whereas the second was performed in June, at 360 days of age, during the feedlot-finishing phase. This design allowed changes in hair-coat characteristics and physiological responses between the two evaluation periods to be assessed.

On each evaluation date, meteorological conditions inside the housing facility were recorded at 10:00 and 15:00 h. The measured variables included air temperature (°C), relative humidity (%), and black-globe temperature (°C). The Radiant Heat Load (RHL; W m⁻²) was calculated from black-globe temperature according to Silva (2008), using the Stefan–Boltzmann equation: RHL = σ × (Tbg + 273.15)⁴, where σ is the Stefan–Boltzmann constant (5.67 × 10⁻⁸ W m⁻² K⁻⁴) and Tbg is black-globe temperature (°C). Rectal temperature was measured at the same times using a digital clinical thermometer inserted directly into the rectum of each animal.

Hair-coat morphological characteristics were assessed on the same evaluation dates at a standardized anatomical site on the flank, approximately 20 cm ventral to the vertebral column. Hair-coat thickness was measured using a caliper inserted perpendicularly to the epidermal surface. At the same anatomical site, a hair sample was removed using duck-bill pliers, placed in an individually labeled plastic bag, and transported to the laboratory.

In the laboratory, hair quantity was determined as the raw number of hairs counted in each collected sample. Numerical hair density was then calculated by standardizing the raw hair count according to the sampled skin area and was expressed as hairs cm⁻². In addition, the ten longest hairs from each sample were visually selected and individually measured using a caliper. The arithmetic mean of these measurements was used to determine mean hair length, following the procedure described by Udo (1978).

### Statistical analyses

**Fig. 1:**
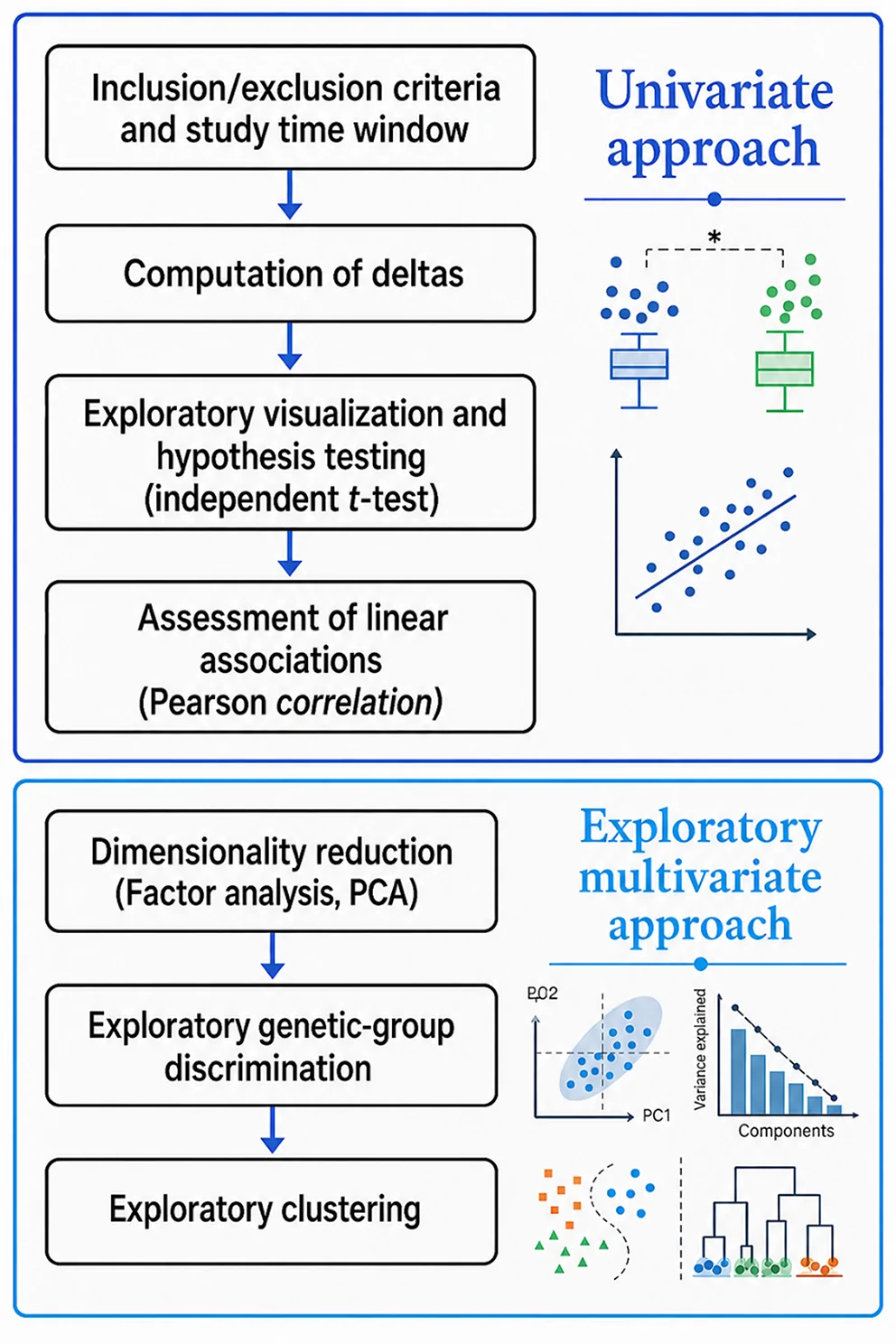
Exploratory analytical workflow used to investigate coat-related thermoregulatory patterns in crossbred cattle

To ensure a clear, reproducible, and interpretable analytical workflow, the statistical strategy followed a structured pipeline integrating univariate inference, dimensionality reduction, exploratory genetic-group discrimination, and unsupervised clustering to investigate coat-related thermoregulatory patterns (Fig. 1). First, inclusion and exclusion criteria and the study time window were defined. Treatment effects were quantified as within-animal changes over time (Δ). For each variable, deltas were calculated as the difference between the last (360 days) and first (210 days) measurements, following recommendations for longitudinal and repeated-measures designs (Festing 2006). For body temperature, daily variation (ΔT) was defined as the difference between mean afternoon and morning rectal temperatures.

Prior to analysis, inferential assumptions were systematically evaluated. Univariate outliers were screened using boxplots, z-scores (|z| > 3), and the interquartile-range criterion. Observations below Q1 − 1.5 × IQR or above Q3 + 1.5 × IQR were considered potential outliers. Multivariate outliers were assessed using Mahalanobis distance with multiple-testing correction. Normality was verified using the Shapiro–Wilk test and visual inspection of histograms and Q–Q plots, while homogeneity of variances between groups was assessed using Levene’s test.

Exploratory visualization and hypothesis testing were then performed. Differences between genetic groups in morphophysiological traits and temperature variables were evaluated using independent-samples Student’s t-tests. Tested variables included Δ coat thickness, Δ hair length, Δ hair quantity, Δ hair density, and morning and afternoon rectal temperatures. Variable distributions were visualized using violin plots generated with Seaborn and Matplotlib, with complementary interactive graphics produced using Plotly.

Linear associations among morphophysiological traits were examined using Pearson correlation coefficients, considering coat traits measured at 360 days of age and daily temperature variation (ΔT). This step provided an initial assessment of pairwise relationships and potential compensatory mechanisms among traits.

To uncover latent structures associated with thermoregulatory adaptation and to reduce data dimensionality, an exploratory factor analysis (EFA) was conducted. Sampling adequacy was explored using the Kaiser–Meyer–Olkin (KMO) statistic (> 0.55) and Bartlett’s test of sphericity (*p* < 0.001). Given the limited sample size, the EFA was interpreted as an exploratory dimensionality-reduction procedure rather than as confirmatory evidence of a stable latent structure. Variables exhibited communalities > 0.60, indicating that, within this exploratory solution, a substantial proportion of their variance was represented by the retained factors. Factor extraction was performed using exploratory factor analysis, and factors were subsequently rotated to improve interpretability. Biological interpretation of the latent constructs was based on the rotated component (loading) matrix, allowing correlated morphophysiological variables to be exploratively summarized into components with potential biological relevance to thermoregulatory mechanisms.

In parallel, Principal Component Analysis (PCA) was applied to standardized adult (data collected at 360 days) coat traits and daily temperature variation to support multivariate visualization and interpretation. PCA scores were subsequently used as the input space for unsupervised clustering.

To explore discrimination between the two genetic groups, binary logistic regression models were fitted within a generalized linear modeling framework using a binomial distribution and a logit link function. Let Y_i_ denote the binary response for animal i, with Y_i_ = 1 for ¾ Red Angus × ¼ Nelore and Y_i_ = 0 for ½ Red Angus × ½ Nelore, and let π_i_ = P (Y_i_ = 1 | X_i_) represent the probability of classification into the target group given the predictor vector X_i_. Three alternative predictor sets were examined: (i) longitudinal changes in coat and physiological variables, including Δ thickness, Δ length, Δ quantity, Δ density, and Δ rectal temperature; (ii) latent factor scores derived from exploratory factor analysis; and (iii) the original environmental, physiological, and coat-related variables. Within each predictor set, stepwise variable selection using a significance threshold of *p* < 0.05 was applied exclusively as an exploratory screening procedure to identify candidate multivariable patterns. Given the small sample size and the consequent risk of model overfitting, these models were not intended for prediction, clinical classification, or confirmatory inference. Model discrimination was summarized using receiver operating characteristic (ROC) curves and the area under the curve (AUC). AUC values close to 0.5 indicate discrimination similar to chance, whereas values approaching 1 indicate stronger apparent discrimination. The reported AUC values represent apparent in-sample discrimination rather than validated predictive performance, and regression coefficients and odds ratios should therefore be interpreted only as hypothesis-generating associations. Model coefficients were expressed as odds ratios (OR), calculated as OR = exp(β).

To identify exploratory coat-related thermoregulatory patterns beyond genetic-group averages, unsupervised clustering analyses were conducted in PCA space. Two complementary methods were applied: agglomerative hierarchical clustering using Ward’s linkage with Euclidean distance, and K-means clustering.

The two methods were applied to explore the sensitivity of the clustering structure to different partitioning procedures. Agglomerative hierarchical clustering captures nested data structure, whereas K-means seeks to minimize within-cluster dispersion. Agreement between the resulting solutions was evaluated descriptively, without assuming equivalence between their cluster labels.

Cluster quality was assessed using the Silhouette Score, which ranges from − 1 to + 1, with higher values indicating better cluster separation. For K-means, WCSS was obtained from the inertia attribute, while for hierarchical clustering it was computed using Euclidean distances. Prior to clustering, all variables were standardized using z-score transformation. After clustering, cluster-wise means were calculated on the original data scale. Finally, the relative contribution of each variable within each cluster was classified as Very Low, Low, Moderate, or High based on variable-specific quartiles (Q1, Q2, Q3) estimated from the global empirical distribution. Cluster means were categorized as Very Low (≤ Q1), Low (Q1–Q2], Moderate (Q2–Q3], or High (> Q3). 

## Results and discussion

### Univariate analyses

**Fig. 2:**
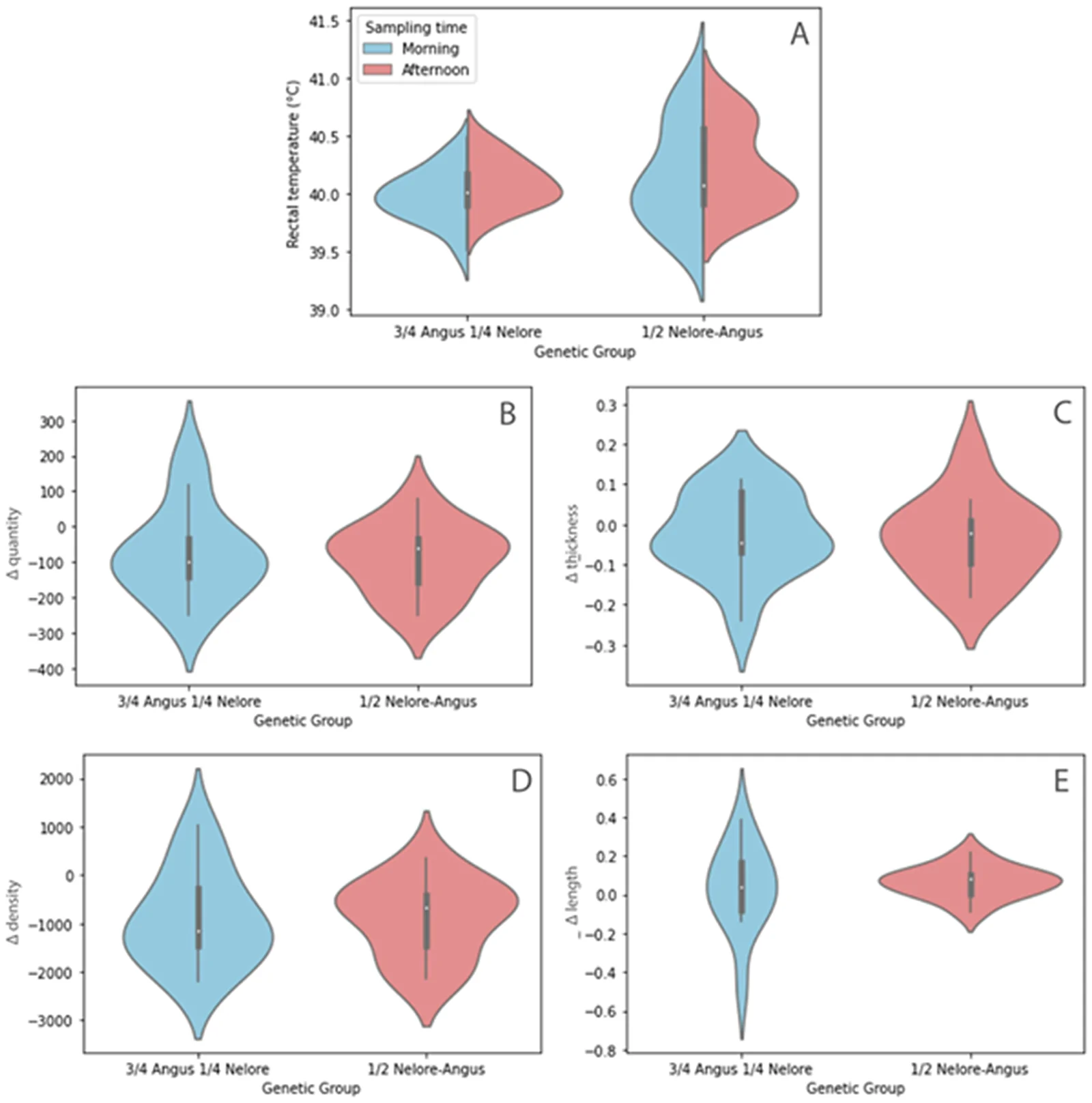
Violin plots comparing rectal temperature and changes in coat traits between genetic groups (¾ Angus ¼ Nelore and ½ Nelore ½ Angus). Note: (**A**) Rectal temperature recorded in the morning (blue) and in the afternoon (red). (**B**–**E**) Changes in coat quantity (**B**), thickness (**C**), density (**D**), and length (**E**) between groups throughout the experiment

No statistically significant differences *(p* > 0.05) were detected between genetic groups for any of the evaluated variables (Δ coat thickness, Δ hair length, Δ hair amount, Δ hair density, or mean rectal temperature) (Table 1; Fig. 2). Accordingly, under the univariate analytical framework, no evidence of between-group differences was detected for the evaluated morphophysiological traits.

**Table 1:** Mean values, standard error and Student’s t test for coat traits and rectal temperature (*n* = 30 bulls)

Variables (units)	Mean	Standard Error	t statistic	*p*-value
Morning rectal temperature (^o^C)	40.08	0.06	-1.47	0.15
Afternoon rectal temperature (^o^C)	40.16	0.53	-1.33	0.19
Δ Thickness* (cm)	-0.03	0.02	0.16	0.88
Δ Length (cm)	0.05	0.03	-0.47	0.64
Δ Quantity (count)	-81.78	23.54	0.32	0.75
Δ Density (cm^− 2^)	-889.78	182.40	-0.01	0.99

*deltas (∆) denote the difference of a given variable between two data collection campaigns (360 days minus 210 days)

**Fig. 3:**
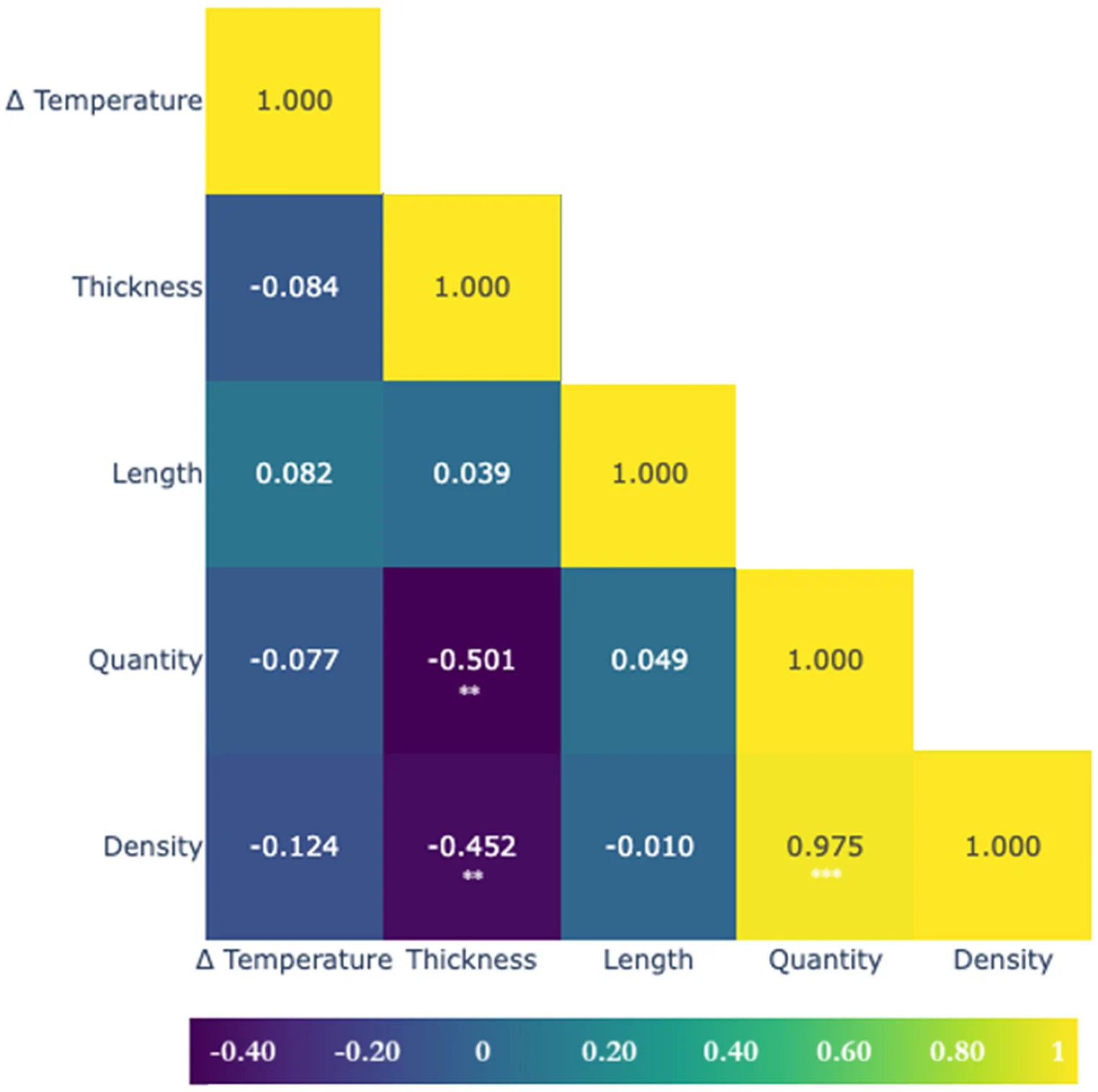
Pearson correlation matrix between animals’ daily change in core temperature (Δ Temperature, computed as rectal temperature in the afternoon minus morning) and end-of-trial coat traits (Thickness, Length, Quantity, and Density). Color saturation represents the correlation magnitude (− 1 to + 1). **P<0.01 *** *P* < 0.001

Pearson correlations (Fig. 3) clarify two-by-two trait interdependencies: hair quantity and density are strongly and positively associated, whereas thickness correlates negatively with both. Together with Fig. 2, this pattern supports a compensatory mechanism: thicker coats tend to present a smaller hair count (Pearson correlation = -0.45, *p* < 0.01) and be less dense (Pearson correlation = -0.50, *p* < 0.01), while abundant hair count coexists with higher density (Pearson correlation = 0.98, *p* < 0.001)—alternative routes to achieve thermoregulation. This compensatory pattern is also characteristic of crossbred bulls (½ Nelore × ½ Angus group), which commonly exhibit intermediate or adaptive combinations of these coat traits.

### Multivariate structure (Factors and PCA)

To probe latent structure, a logistic model with Δ variables was first tested (Table 2), but no statistically significant associations emerged; a stepwise procedure failed as well to identify significant combinations (*p* < 0.05), precluding a stable convergent solution. Complementarily, factor analysis (Table 3) produced a variable ranking whose correlation with genotypic groups was weak and negative—insufficient to indicate clear separation.

**Table 2:** Coefficients and p-values of the variables used in the logistic model to predict the genetic group of the animals (*n* = 30 bulls)

Variables	Coefficient	*p*-value
Δ Rectal temperature	0.3939	0.481
Thickness	-0.5638	0.337
Length	-0.7002	0.193
Quantity	-2.2145	0.371
Density	0.6311	0.787
Intercept	-0.2323	0.654

Note: The model was fitted assuming a binomial distribution for the response variable, with a *Log-Likelihood* of -12.286 and a *Pseudo R²* of 0.2710. The table reports the estimated coefficients and *p*-values for each variable, along with the statistical significance of each predictor in the initial model. None of the predictors showed a *p*-value lower than the adopted significance level (0.05)

**Table 3:** Pearson correlation matrix between Ranking and Genotype variables (*n* = 30 bulls)

	Ranking	Genotype
Ranking	-	
Genotype	-0.2921	-

**Fig. 4:**
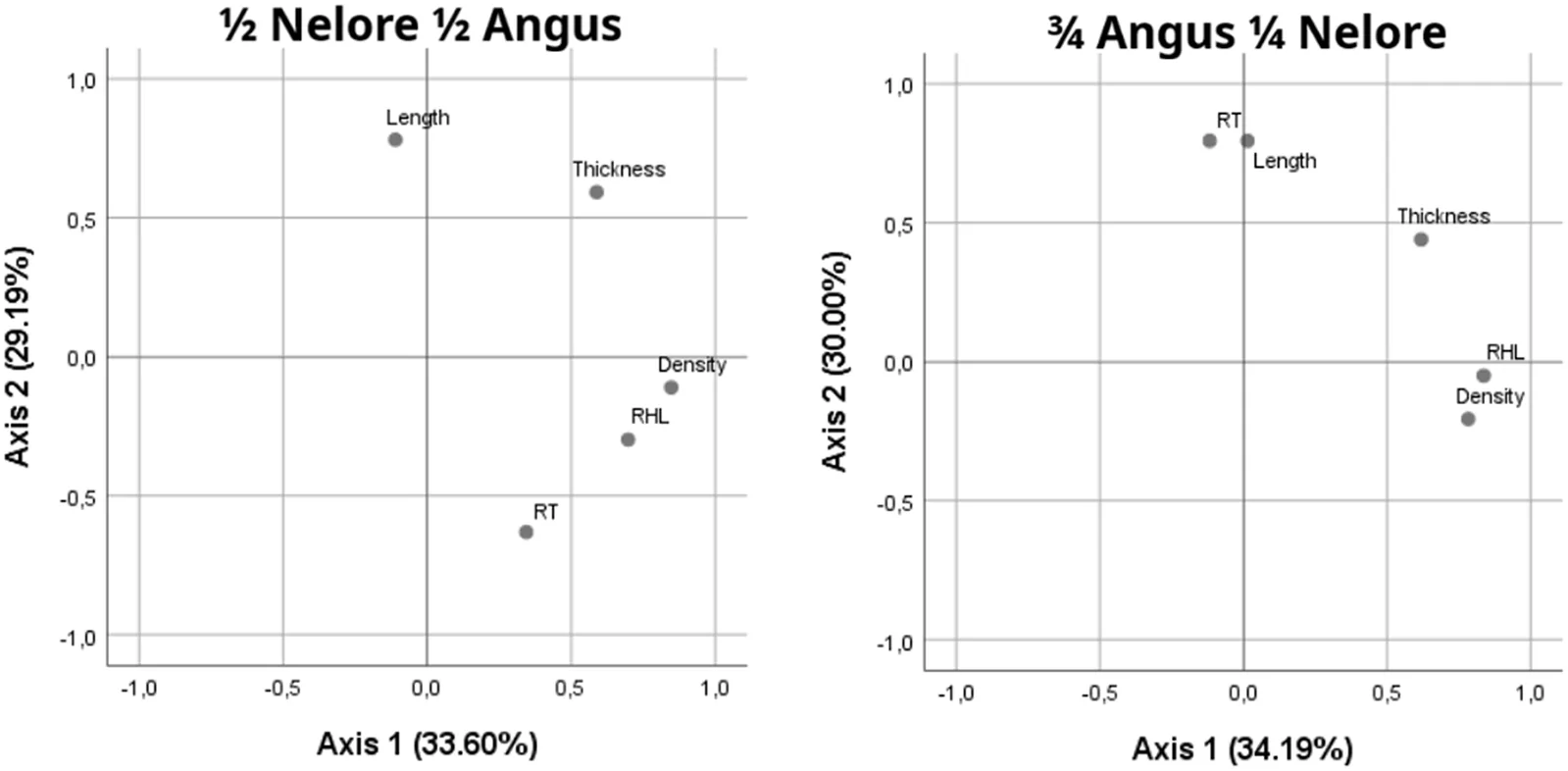
Factor Analysis by genotype — ½ Nelore × ½ Angus (left) and ¾ Angus × ¼ Nelore (right). Dots depict factor loadings of the variables on the first two factors (Axis 1 and Axis 2), with explained variance shown in parentheses on each axis. Variables: Length, Thickness, Density, RHL (radiant heat load, W. m²), and RT (rectal temperature, °C). Note: Greater distance from the origin denotes a stronger contribution of the variable to the factor; the sign indicates loading direction. Comparing panels suggests subtle genotype-specific latent structures (e.g., closer association between RT and Length in ¾ Angus × ¼ Nelore). Axis percentages indicate the common variance explained by each factor

Principal Component Analysis provided a coherent multivariate picture. In both crossbreds, the first two axes explained ~ 63–64% of the variance (Axis 1 ≈ 33–34%; Axis 2 ≈ 29–30%; Fig. 4). Some differentiation between crossbreds was observed along PC2. In ½ Nelore × ½ Angus (Fig. 4, left panel), hair length loaded strongly and positively, whereas rectal temperature (RT) loaded negatively, suggesting a decoupling between coat length and body heat load. This interpretation is consistent with the literature. For example, Davila et al. (2019) demonstrated that, for every 25% increase in Brahman composition, undercoat length, topcoat length, core body temperature under low temperature–humidity index (THI), and core body temperature under high THI decreased by 1.32 mm, 2.94 mm, 0.11 °C, and 0.14 °C, respectively. These findings confirm that crossbreds with greater zebuine influence develop shorter coats and maintain lower body temperatures (Façanha et al. 2019; Silveira et al. 2021).

In ¾ Angus × ¼ Nelore (Fig. 4, right panel), RT appeared close to hair length in the upper quadrant, indicating a direct association—longer coats were positioned in the same multivariate direction as higher RT values, suggesting an association between coat length and rectal temperature within this exploratory ordination. These patterns align with reports showing that animals with higher proportions of Angus (and reduced zebuine fraction) exhibit the longest coat lengths and the highest body temperatures under heat stress. The literature consistently documents that European/taurine breeds, when exposed to thermal challenge, exhibit higher rectal temperatures than zebuine cattle and their crossbreds, reinforcing that long and dense coats elevate thermal load (Brown-Brandl 2018).

Hair thickness remained positive on both axes, indicating an ambiguous role: on one hand, greater thickness can shield against solar radiation without increasing RT when combined with low hair density—a strategy commonly reported in zebuine breeds (Gebremedhin et al. 2023). On the other hand, when associated with high density, thickness aligns with the radiant heat load gradient, hindering heat dissipation. Recent reviews emphasize that haircoats act as insulators by trapping air, but at the same time may impede sweating efficiency, and that long, dense coats retain sweat and reduce convective heat loss (Brown-Brandl 2018). Although sweat-gland morphology was not measured directly in the present study, the combined effects of coat length, density, and thickness may be related to variation in sweating capacity and sweat-gland characteristics reported in the literature. For example, Brahman cattle have been reported to possess larger and more numerous sweat glands than taurine breeds, resulting in greater sweating capacity (Mateescu et al. 2023). Thus, this mechanism should be regarded as a biologically plausible interpretation rather than as a directly measured explanation for the patterns observed here.

In summary, the multivariate interpretation of the exploratory factor analysis aligns closely with the literature: crossbreds with greater zebuine influence (shorter coats) show enhanced ability to maintain lower body temperatures even when coat thickness increases, whereas those with greater taurine influence (long and dense coats) exhibit proportionally higher thermal load. This body of evidence reinforces that thermal biology emerges from the integrated interaction among coat length, density, thickness, and sweating capacity, explaining the separation patterns observed between groups in the multivariate analysis. Consequently, individual traits cannot be interpreted in isolation; thermophysiological responses operate as systemic, interconnected processes.

### Exploratory genetic-group discrimination

**Fig. 5:**
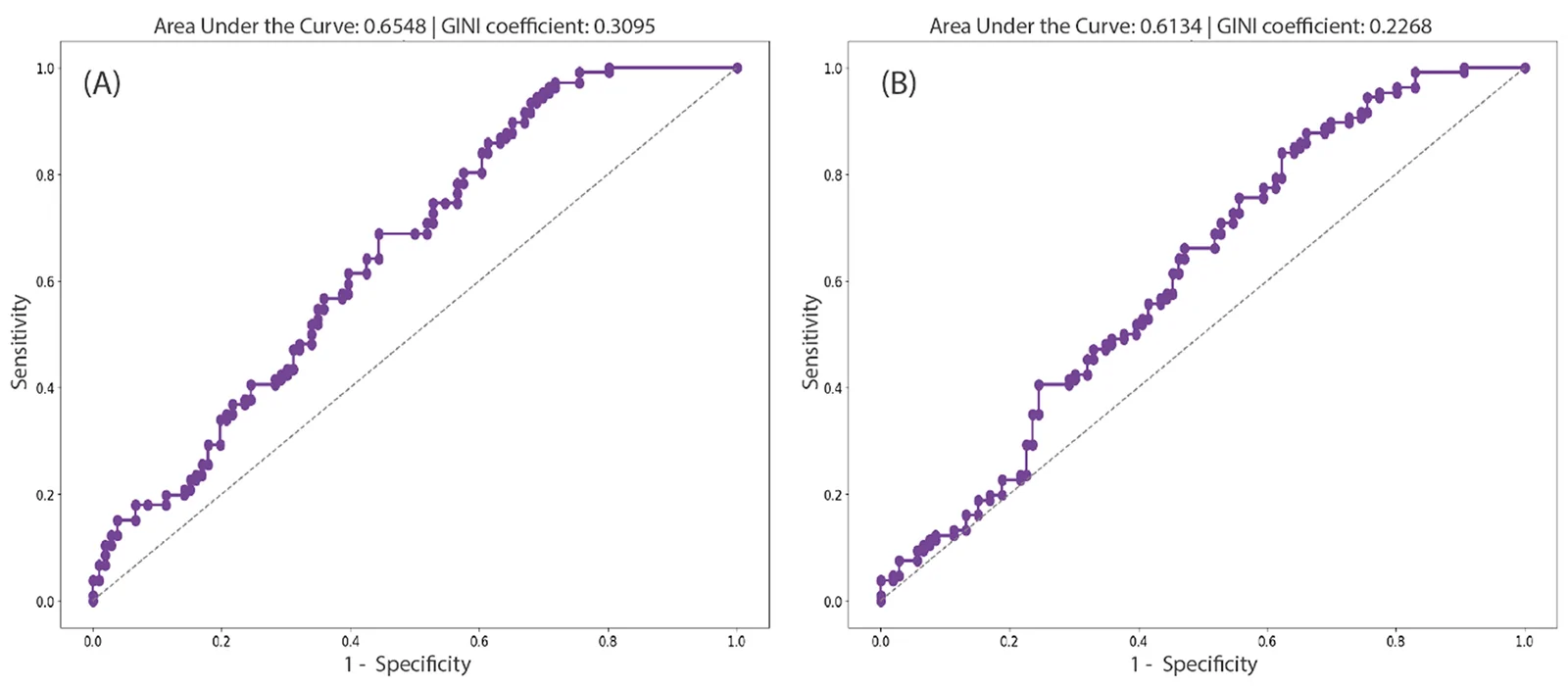
ROC curves showing the performance of two logistic regression models: (**A**) built from environmental, temperature, and coat variables; (**B**) built from factor-analysis–derived latent factors. Note: The areas under the curve (AUC) were 0.6548 and 0.6134, indicating moderate, above-chance discrimination. The corresponding Gini coefficients were 0.3095 and 0.2268 (computed as Gini = 2×AUC − 1, corroborating the models’ moderate efficacy. Sensitivity (true-positive rate) is plotted against 1 − specificity (false-positive rate), illustrating trade-offs across thresholds

Beyond structure, we assessed discriminative ability. Two logistic regressions were fitted (Tables 4 and 5), one with original variables and the other with latent factors; both showed overall moderate performance (AUC > 0.5; Fig. 5). Despite different specifications, ROC curves were similar, indicating that individual variables and consolidated factors capture a common underlying signal.

**Table 4:** Binary logistic regression model using the original variables (*n* = 30 bulls)

Morphophysiological response	Coefficient	Odds ratio	*p*-value
Intercept	-0.0101	0.9900	= 0.945
Rectal temperature	0.3434	1.4097	= 0.023
Length	-0.6414	0.5266	< 0.001

**Table 5:** Binary logistic regression model with factors derived from factor analysis

Variables	Coefficient	Odds ratio	*p*-value
Intercept	-0.0018	0.9982	= 0.990
Factor 1	-0.4932	0.6107	< 0.001

In the exploratory binary logistic regression based on morphophysiological variables (Table 4), rectal temperature (β = 0.3434; OR = 1.41; *p* = 0.023) and hair length (β = −0.6414; OR = 0.53; *p* < 0.001) were associated with the probability of classification into the target genetic group. Conditional on the model and the adopted group coding, a one-unit increase in rectal temperature was associated with a 41% increase in the odds of classification into the target group, whereas a one-unit increase in hair length was associated with a 47% reduction in those odds. These associations should be interpreted as exploratory, in-sample group-discrimination signals rather than as validated predictors of heat tolerance. Although rectal temperature and coat morphology are biologically relevant indicators of thermoregulatory responses in cattle (Sejian et al. 2018; Amorim et al. 2019; Mascarenhas et al. 2023), the present analysis does not demonstrate that either genetic group is intrinsically more heat tolerant or more susceptible to heat stress. Rather, the findings suggest that physiological and morphological characteristics may jointly contribute to the observed differentiation between the two genetic compositions.

### Exploratory multivariate phenotyping

**Fig. 6:**
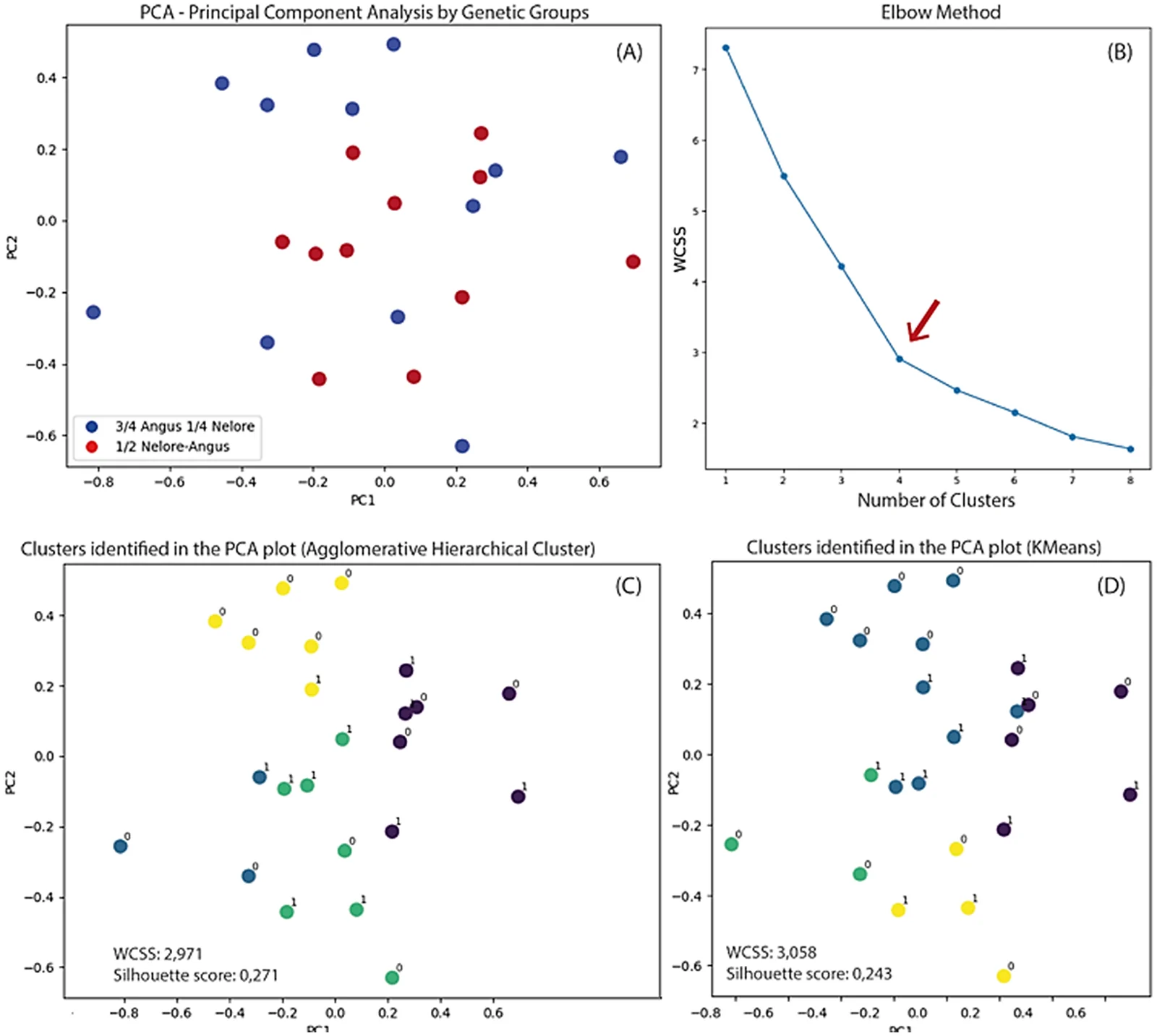
(**A**) Principal Component Analysis (PCA) based on coat traits (quantity, density, thickness, and length) and the average daily change in core temperature (ΔT = afternoon − morning). (**B**) Elbow method to select the optimal number of clusters using within-cluster sum of squares (WCSS). (**C**) Agglomerative hierarchical clustering plotted on the PCA axes. (**D**) K-means clustering plotted on the PCA axes. Note: (**A**) Points are colored by original genetic group. (**B**) The red arrow marks the point where WCSS reduction becomes marginal, supporting the chosen number of clusters. (**C**–**D**) Clustering quality was assessed using the Silhouette Score (0.271 and 0.243) and Inertia/ WCSS (2.971 and 3.058), respectively. Each point represents one individual; genetic labels are shown next to markers: 0 = ¾ Angus × ¼ Nelore; 1 = ½ Nelore × ½ Angus

To uncover substructures obscured by group overlap, PCA scores served as the basis for clustering (Fig. 6). The Elbow Method guided the choice of cluster numbers (Fig. 6B). Agglomerative hierarchical clustering (Ward’s method; Fig. 6C) and K-means clustering (Fig. 6D) produced broadly similar spatial arrangements, with differences occurring mainly among a small number of borderline individuals, indicating partial agreement between the two exploratory clustering solutions. Loadings (Table 6) indicate that PC1 reflects thickness versus quantity/density, while PC2 tracks hair length and daily thermal variation.

**Table 6:** Loadings of the original variables on the principal axes generated by Principal Component Analysis (PCA)

Variables	PC1	PC2
Thickness	0.766	-0.050
Length	0.057	0.915
Quantity	-0.472	0.038
Density	-0.432	-0.009
Δ Rectal temperature	-0.001	0.399

The exploratory clustering solutions suggested four candidate coat-related configurations. Two candidate configurations showed lower daily rectal-temperature variation, although they were characterized by distinct coat morphologies:

- Purple: thick coats that are less abundant and less dense;
- Blue: fine, short, dense, abundant coats.

This contrast supports a compensatory hypothesis: different coat architectures can contribute to thermal stability. The blue cluster’s success aligns with Farias et al. (2024), who reported greater heat tolerance in Angus with short, fine hair under tropical conditions. Both profiles maintain homeothermy—vital to prevent heat stress—consistent with literature describing insulation- versus dissipation-driven routes to thermal stability.

Two other clusters exhibited greater daily thermal variation (lower acclimation efficiency), but for opposite reasons:

- Green: sparse, short hair of moderate density—possibly insufficient insulation combined with only partial dissipation;
- Yellow: long, abundant, very dense hair—strong insulation useful at night/cold periods but hindering heat loss during hot hours.

Compensatory strategies (e.g., increased respiratory rate) may be engaged in the latter. The yellow cluster was dominated by ¾ Angus × ¼ Nelore, consistent with a heavier coat and lower heat-loss capacity typical of taurine breeds (McManus et al. 2009). This duality is in concordance with Barreto et al. (2024): thick, long, larger-diameter, dense coats protect against solar radiation but hinder heat dissipation, whereas fine, short, less-dense coats facilitate heat loss but offer less solar protection. The hierarchical and K-means analyses both suggested four exploratory clusters, but the solutions differed in the allocation of some individuals and in the configurations associated with their method-specific labels. Therefore, the clusters should not be interpreted as directly equivalent across methods. The variables characterizing each solution are summarized separately for the hierarchical clustering in Table 7 and for K-means in Table 8. Given the observed overlap between clusters, these configurations should be regarded as exploratory candidate patterns rather than as stable or validated phenotypic groups.

**Table 7:** Classification of variables in each cluster generated by the agglomerative hierarchical model, based on variable loadings from Principal Component Analysis (PCA)

Methods	Purple	Blue	Green	Yellow
Thickness	High	Very low	Moderate	Low
Length	Moderate	Moderate	Low	Very High
Quantity	Low	High	Moderate	Moderate
Density	Low	High	Moderate	Moderate
Δ rectal temperature	Moderate	Very low	High	Very high

**Table 8:** Classification of variables within each K-means cluster, based on variable weights (loadings) derived from Principal Component Analysis (PCA)

Methods	Purple	Green	Blue	Yellow
Thickness	High	Low	Very Low	Moderate
Length	Moderate	Moderate	Low	Very Low
Quantity	Very Low	Moderate	High	Low
Density	Very Low	Moderate	High	Low
Δ Rectal temperature	Low	High	Very Low	Low

Cluster-validity metrics indicated weak separation and partial overlap between the exploratory clustering solutions. Silhouette scores were low for both methods, although the hierarchical solution showed slightly better separation than K-means (0.271 vs. 0.243). Because the WCSS values were obtained from different clustering procedures, they were not used to establish the superiority or greater compactness of either method. Overall, these findings indicate that the identified configurations should be interpreted as exploratory candidate patterns rather than as robust, stable, or validated phenotypic groups.

Recent research has demonstrated the potential of artificial intelligence for large-scale heat-tolerance phenotyping. Chlingaryan et al. (2025), for example, combined approximately three million dairy production records with 20 years of climatic data and integrated artificial intelligence with a statistical genetic model. Heat tolerance was represented by the individual slope of milk-yield decline when the temperature–humidity index exceeded 60. In contrast, the present study uses direct, low-cost measurements of coat morphology and rectal temperature in a small group of beef crossbreds. Therefore, our contribution is exploratory and mechanistic rather than predictive: we examine candidate combinations of coat traits associated with rectal-temperature variation, without estimating breeding values or validated heat-tolerance phenotypes. These approaches are complementary in scale and purpose, but the patterns identified here require validation in larger independent populations.

## Implications

The substantial overlap observed between the genetic groups, together with the limited apparent in-sample discrimination of the logistic models, reinforces an important implication for the thermal physiology of production animals: thermoregulatory responses are multivariate and context-dependent. Univariate analyses remain useful for identifying general tendencies and pairwise associations; however, they should be considered complementary to multivariate analyses rather than as independent alternatives. By integrating coat morphology, physiological responses, and changes over time, the exploratory multivariate framework used in this study provided a more comprehensive representation of compensatory relationships that may remain obscured when traits are evaluated separately. In particular, the combined use of longitudinal changes, correlation analysis, dimensionality reduction, exploratory genetic-group discrimination, and unsupervised clustering helped identify candidate coat-related thermoregulatory configurations. These patterns may contribute to a better biological understanding of thermal responses in crossbred cattle, but their practical relevance for phenotypic screening, breeding, or management decisions requires confirmation in larger and independent populations.

## Limitations

This study provides exploratory insights into the relationships between coat characteristics and thermoregulatory responses in Red Angus × Nelore crossbred cattle; however, several limitations must be acknowledged. First, the analyses were based on a relatively small sample of 30 clinically healthy bulls, which limits statistical power and the generalizability of the findings. Although model assumptions were evaluated, the limited sample size increases uncertainty around the logistic-regression estimates and the stability of the clustering solutions.

Second, the study focused primarily on coat morphology and rectal-temperature variation and did not include other physiological indicators, such as respiratory rate, sweating rate, or skin temperature. Productive outcomes, including weight gain, feed efficiency, carcass characteristics, and meat quality, were also not evaluated. Consequently, the study does not establish whether the identified morphophysiological patterns are associated with productive resilience or validated heat-tolerance phenotypes. In addition, the absence of purebred Nelore and Red Angus reference groups prevented direct quantification of how the observed crossbred phenotypes compare with the thermal and coat-related characteristics of their parental breeds.

Finally, the logistic-regression and clustering analyses were exploratory and were not externally validated. The reported AUC values represent apparent in-sample discrimination, while the low Silhouette Scores indicate weak separation and partial overlap among the clustering configurations. Therefore, the identified patterns should be interpreted as candidate thermoregulatory configurations rather than as stable or validated phenotypic groups. Future studies should include larger samples, independent herds, purebred parental reference groups, contrasting environmental conditions, repeated physiological measurements, and productive and economic indicators to assess the consistency and broader applicability of these exploratory findings.

## Conclusion

In summary, the two Red Angus × Nelore genetic groups showed substantial similarity in individual coat traits and rectal temperature under the evaluated tropical semi-arid conditions. However, the integrated multivariate approach revealed biologically meaningful combinations of coat characteristics associated with daily rectal-temperature variation. Coat thickness, hair length, hair quantity, and hair density acted as an interconnected system, indicating that different integumentary configurations may contribute to thermal regulation through alternative balances between insulation and heat dissipation.

The exploratory analyses suggested four candidate coat-related thermoregulatory configurations. Short, fine, and dense coats and thicker but less dense coats were associated with lower daily rectal-temperature variation, whereas intermediate and long, dense coats were associated with greater thermal fluctuation. These findings highlight the value of multivariate phenotyping for detecting compensatory relationships that may remain obscured when traits are evaluated individually. The identified patterns provide a biologically grounded framework for understanding coat-related thermal responses in tropical crossbred cattle and warrant validation in larger, independent populations and under different environmental conditions.

## Data Availability

The datasets generated and/or analyzed during the current study are available from the corresponding author upon reasonable request.

## References

[CR1] Alvares CA, Stape JL, Sentelhas PC, de Moraes Gonçalves JL, Sparovek G (2013) Köppen’s climate classification map for Brazil. Meteorol Z 22(6):711–728. 10.1127/0941-2948/2013/0507

[CR2] Amorim MLCM, Saraiva EP, de França Carvalho Fonsêca V, Guerra RR, Santos D, de Melo Costa SGCG, Almeida CC, da Costa MEV, Pinheiro A, Filho P, E.C (2019) Coat and skin morphology of hair sheep breeds in an equatorial semi-arid environment. J Therm Biol 84:103–110. 10.1016/j.jtherbio.2019.06.007 31466742

[CR4] Brown-Brandl TM (2018) Understanding heat stress in beef cattle. Revista Brasileira de Zootecnia 47:e20160414. 10.1590/rbz4720160414

[CR5] Camargo LSA, Saraiva NZ, Oliveira CS, Carmickle A, Lemos DR, Siqueira LGB, Denicol AC (2023) Perspectives of gene editing for cattle farming in tropical and subtropical regions. Anim Reprod 19(4):e20220108. 10.1590/1984-3143-AR2022-0108 PMC9924776 36819485

[CR6] Chlingaryan A, Thomson PC, Garcia SC, Clark CEF (2025) An AI-based hybrid model for dairy cattle heat tolerance phenotype. Smart Agricultural Technol 12:101455. 10.1016/j.atech.2025.101455

[CR7] da Silva WST, Silveira RMF, da Silva WE et al (2025) Coat traits, skin evaporation, and milk production of Majorera and Palmera goats in a temperate region. Int J Biometeorol 69:3167–3181. 10.1007/s00484-025-03016-0 41023253

[CR31] Davila KMS, Hamblen H, Hansen PJ, Dikmen S, Oltenacu PA, Mateescu RG (2019) Genetic parameters for hair characteristics and core body temperature in a multibreed Brahman-Angus herd1. J Anim Sci 97(8):3246–3252. 10.1093/jas/skz188 PMID: 31214688; PMCID: PMC6667242 PMC6667242 31214688

[CR8] do Barreto N, Jacintho A, Junior MCB, W., et al (2024) Adaptive integumentary features of beef cattle raised on afforested or non-shaded tropical pastures. Sci Rep 14:16951. 10.1038/s41598-024-66675-w PMC11266545 39043710

[CR10] Façanha DAE, Ferreira JB, Leite JHGM, de Sousa JER, Guilhermino MM, Costa WP, Bermejo Asensio LA, de Vasconcelos AM, Silveira RMF (2019) The dynamic adaptation of Brazilian Brahman bulls. J Therm Biol 81:128–136. 10.1016/j.jtherbio.2019.02.016 30975410

[CR9] FAO (2023) Meat Market Review: Overview of global market developments in 2023. Food and Agriculture Organization of the United Nations, Rome

[CR11] Farias CO, Lazzari J, Soares da Cunha Í, Gonçalves PBD, Gasperin BG, Lucia T, Jr, Schmitt E, Cardoso FF, Sarubbi J, Mondadori RG (2024) Thermotolerance in Angus cattle is related to hair coat characteristics but not to coat color. J Therm Biol 124:103945. 10.1016/j.jtherbio.2024.103945 39142266

[CR12] Festing MFW (2006) Design and statistical methods in studies using animal models of development. ILAR J 47(1):5–14. 10.1093/ilar.47.1.5 16391426

[CR30] Gebremedhin KG, Fonseca VDFC, Maia ASC, Methods (2023) Thermodynamic Applications, and Habitat Implications of Physical and Spectral Properties of Hair and Haircoats in Cattle. Anim (Basel) 13(19):3087. 10.3390/ani13193087 PMID: 37835693; PMCID: PMC10571584 PMC10571584 37835693

[CR13] Hernandez AS, Zayas GA, Rodriguez EE et al (2024) Exploring the genetic control of sweat gland characteristics in beef cattle for enhanced heat tolerance. J Anim Sci Biotechnol 15:66. 10.1186/s40104-024-01025-4 PMC11077762 38715151

[CR17] Mascarenhas NMH, Furtado DA, de Souza BB, de Sousa OB, da Costa ANL, Feitosa JV, da Silva MR, Batista LF, Dornelas KC (2023) Morphology of coat and skin of small ruminants reared in the Brazilian semi-arid region. J Therm Biol 103:103418. 10.1016/j.jtherbio.2022.103418 36796883

[CR32] Mateescu RG, Sarlo Davila KM, Hernandez AS, Andrade AN, Zayas GA, Rodriguez EE, Dikmen S, Oltenacu PA (2023) Impact of Brahman genetics on skin histology characteristics with implications for heat tolerance in cattle. Front Genet 14:1107468. 10.3389/fgene.2023.1107468 PMC10203561 37229201

[CR18] McManus C et al (2009) Heat tolerance in naturalized Brazilian cattle breeds. Livest Sci 120(3):256–264. 10.1016/j.livsci.2008.07.014

[CR19] Michael P, de Cruz CR, Mohd Nor N, Jamli S, Goh YM (2022) The potential of using temperate–tropical crossbreds and agricultural by-products, associated with heat stress management for dairy production in the tropics: A review. Animals 12(1):1. 10.3390/ani12010001 PMC8749655 35011107

[CR20] National Research Council (NRC) (1996) Nutrient Requirements of Beef Cattle, 7th edn. National Academy, Washington, DC

[CR21] Sejian V, Bhatta R, Gaughan JB, Dunshea FR, Lacetera N (2018) Adaptation of animals to heat stress. Animal 12(2):s431–s444. 10.1017/S1751731118001945 30139399

[CR22] Silva RG (2008) Biofísica ambiental: os animais e seu ambiente. FUNEP, São Paulo

[CR24] Silveira RMF, Ferreira J, Busanello M, de Vasconcelos AM, Façanha DAE (2021) Relationship between thermal environment and morphophysiological, performance and carcass traits of Brahman bulls raised on tropical pasture: A canonical approach to a set of indicators. J Therm Biol 95:102814. 10.1016/j.jtherbio.2020.102814 33627260

[CR23] Silveira RMF, Façanha DAE, McManus CM, Ferreira J, da Silva IJO (2023) Machine intelligence applied to sustainability: A systematic methodological proposal to identify sustainable animals. J Clean Prod 420:138292. 10.1016/j.jclepro.2023.138292

[CR26] Silveira RMF, Façanha DAE, McManus C, Asensio LAB, da Silva IJO (2024) Intelligent methodologies: An integrated multi-modeling approach to predict adaptive mechanisms. Comput Electron Agric 216:108502. 10.1016/J.COMPAG.2023.108502

[CR25] Silveira RMF, McManus C, da Silva (2026) I.J.O. Animal production under climate change: a global scientometric analysis of research structure, thematic evolution, and knowledge gaps. Trop Anim Health Prod 58:282. 10.1007/s11250-026-05071-0 PMC13219208 42207432

[CR27] Udo HM (1978) Hair coat characteristics in Friesian heifers in the Netherlands and Kenya: Experimental data and a review of literature. Veenman, Wageningen

